# Clinical course of COVID-19 infection in a melanoma patient treated with nivolumab and bempegaldesleukin: a case report

**DOI:** 10.2217/imt-2021-0331

**Published:** 2022-07-19

**Authors:** Amalia Anastasopoulou, Aikaterini Gkoufa, Panagiotis Diamantopoulos, Spyridon Kazanas, Irene Eliadi, Michael Samarkos, Helen Gogas

**Affiliations:** ^1^First Department of Internal Medicine, Laikon General Hospital, Medical School of National & Kapodistrian University of Athens, Athens, 11527, Greece

**Keywords:** bempegaldesleukin, case report, COVID-19, cytokines, immune-related pneumonitis, immunotherapy, melanoma, nivolumab

## Abstract

The exact impact of immune checkpoint inhibitors in the course and outcome of COVID-19 in cancer patients is currently unclear. Herein, we present the first description of an elderly melanoma patient who developed COVID-19 pneumonia while under treatment with nivolumab and bempegaldesleukin in combination with an investigational PEGylated interleukin (IL-2). We present the clinical characteristics and the laboratory and imaging findings of our patient during the course of COVID-19 pneumonia. Moreover, we discuss the currently available data regarding the mechanism of action of immune checkpoint inhibitors and IL-2 analogs in the treatment of COVID-19. The administration of these agents did not have a negative effect on the outcome of COVID-19 pneumonia in an elderly melanoma patient.

## Plain language summary

Immune checkpoint inhibitors represent a major advance in the treatment of several solid malignancies, including melanoma. Bempegaldesleukin is an investigational PEGylated IL-2 that is being evaluated, in combination with nivolumab, in the management of a variety of cancers. The immunomodulation caused by these agents may also modify the immune response in COVID-19. Currently available data regarding the impact of immune checkpoint inhibitors in reducing the severity of COVID-19 in patients with cancer are mixed, whereas no clinical data are available for bempegaldesleukin. Herein, we report the case of an elderly female melanoma patient who developed COVID-19 pneumonia while under treatment with nivolumab and bempegaldesleukin. The administration of these agents did not have a negative effect on the outcome of COVID-19 pneumonia in our patient.

Coronavirus disease 2019 (COVID-19), caused by severe acute respiratory syndrome coronavirus 2 (SARS-CoV-2), emerged in China in December 2019, and it was soon declared as a pandemic, having major complications such as pneumonia and respiratory failure. The pandemic has resulted in diagnostic delays in cancer patients [1,2]. In addition, the clinical phenotype of COVID-19 in patients with cancer is poorly described, and it is unknown whether the presence of malignancy *per se* is associated with a complicated course of COVID-19 due to the heterogeneity among different studies with regard to patient characteristics (such as age, frailty and presence of comorbidities) as well as cancer type/stage and treatment [3]. Moreover, the available data regarding the impact of cancer treatment in the outcome of COVID-19 are mixed [4–9]. Immune checkpoint inhibitors (ICIs) have revolutionized oncology, but they might also have a protective and therapeutic role in viral infections and thus are of great interest in the era of COVID-19 [10,11]. Recently, the efficacy and safety of the combination of nivolumab, an ICI targeting the PD-1, with bempegaldesleukin (NKTR-214), an investigational PEGylated IL-2 acting as a CD122-preferential IL-2 pathway agonist, in patients with advanced solid cancers has been explored in a phase I dose-escalation study [12] and several phase III clinical trials are currently in progress. In this study, we describe an elderly melanoma patient who presented with COVID-19 while on treatment with nivolumab and bempegaldesleukin and discuss the potential impact of these agents on the clinical course of the infection in our patient.

## Case presentation

**Figure 1.**
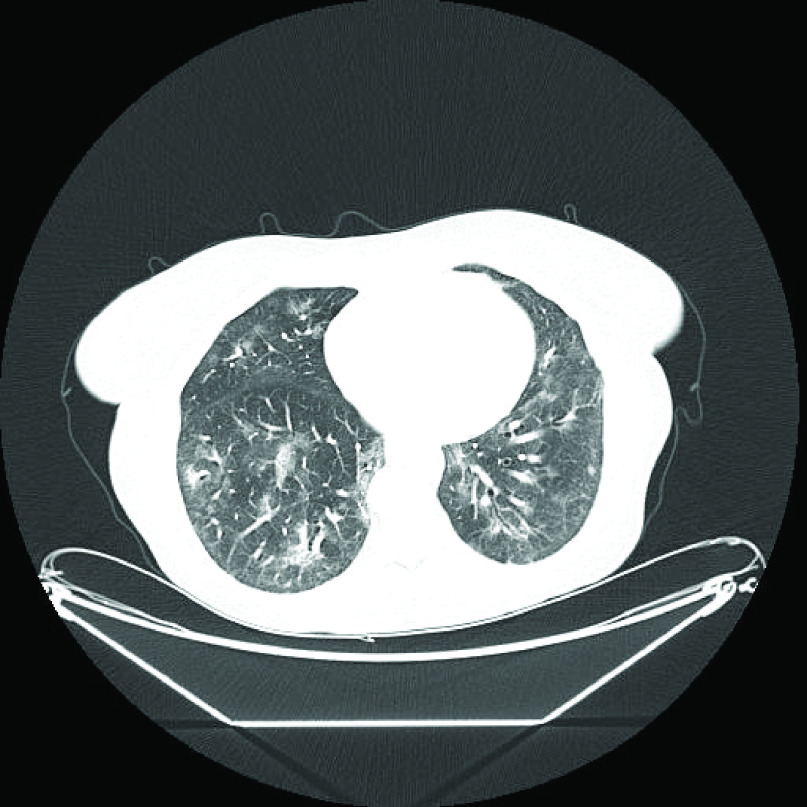
Chest CT performed upon COVID-19 diagnosis showing multiple ground-glass opacities and patchy consolidations in both lungs.

**Figure 2.**
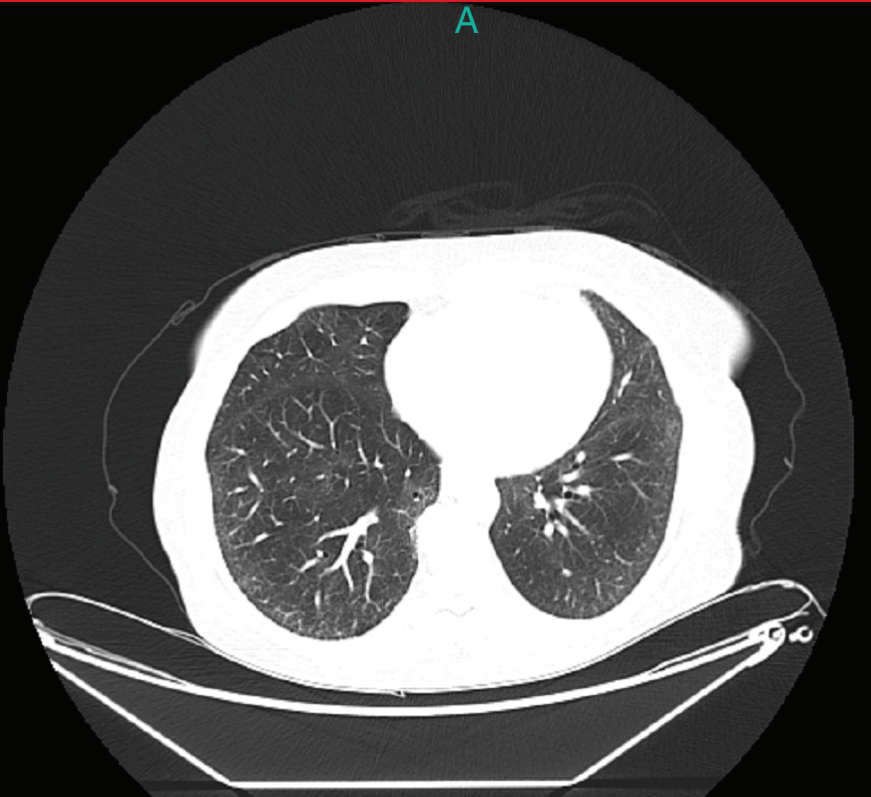
Follow-up chest CT performed 1 month after COVID-19 diagnosis showing almost complete resolution of prior lung infiltrates.

**Figure 3.**
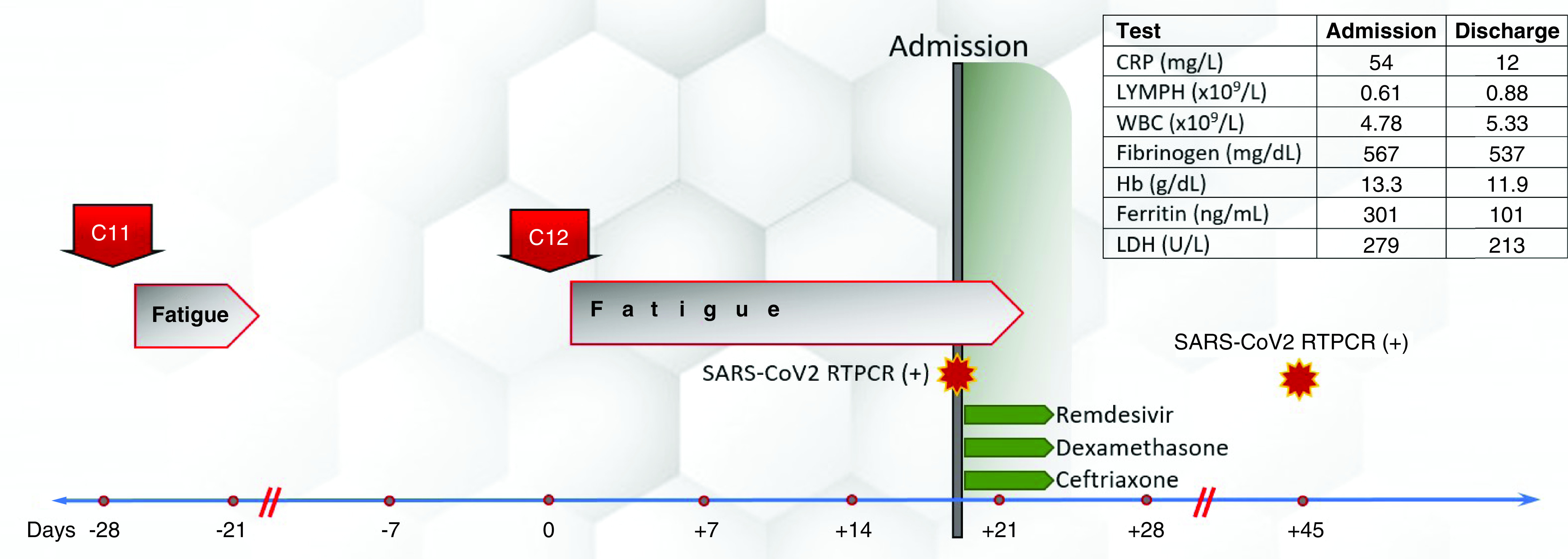
Clinical course of COVID-19 pneumonia in a melanoma patient under treatment with nivolumab and bempegaldesleukin.

An 82-year-old female patient with a history of hypothyroidism underwent an excisional biopsy of a non-pigmented lesion of the left sole. Histology demonstrated a non-ulcerated malignant melanoma with vertical and horizontal growth phase. Breslow depth was 2.4 mm, mitotic index was 8/mm^2^, and the lymphocytic response was non-brisk. Subsequently, a wide local excision was performed, and no residual tumor cells were found. Moreover, histology of a sentinel left inguinal lymph node was negative (stage IIA melanoma, T3aNoMo). In addition, *BRAF* mutation status analysis did not detect *BRAF* mutations. 9 months later, the patient experienced a recurrence located in her left sole that was surgically removed and 10 months later she had a relapse in left external iliac and inguinal lymph nodes (M1a, stage IV). She underwent a lymph node dissection and upon computed tomography (CT) restaging a subcutaneous lesion was identified. She was then enrolled in a randomized, phase III, open-label study of NKTR-214 with nivolumab versus nivolumab alone (CA 045-001) and was randomized to receive NKTR-214 (0.46 mg Q3W) plus nivolumab (360 mg Q3W). After cycle 5 and after each infusion the patient experienced grade 2 fatigue that would resolve spontaneously within up to 7 days. Three months after treatment initiation she achieved a complete remission (CR). After the 12th cycle of treatment, the patient reported fatigue as with previous infusions, which, however, did not resolve in the following three weeks. Upon examination, three weeks after the 12th infusion, she had an axillary temperature of 36.1°C, her blood pressure was 110/70 mmHg, her pulse 110 bpm, her oxygen saturation 96% at rest on ambient air, and her respiratory rate 15 breaths per minute. However, she experienced oxygen desaturation (91%) and dyspnea during mild exertion. Lung auscultation and the rest of the clinical examination were normal. Laboratory tests were normal, except for lymphopenia (0.61 x 10^9^/l) and a mild elevation of C-reactive protein (CRP), at 54 mg/l (normal range: 0–5). A qualitative SARS-CoV-2 real-time reverse transcription polymerase chain reaction (RT-PCR) on a nasopharyngeal swab was positive. The patient was unvaccinated against SARS-CoV-2 because vaccines were not available at that time. Immediately after diagnosis, the patient's husband was also screened for SARS-CoV-2 and tested positive. A chest CT revealed diffuse bilateral and peripheral lung infiltrates with less than 50% parenchymal involvement, suggestive of a viral infection (Figure 1). She was admitted and received dexamethasone (6 mg q24h), remdesivir (loading dose of 200 mg followed by 100 mg q24h), and empiric antimicrobial treatment with ceftriaxone at 2 gr q24h as well as oxygen supplementation through a nasal cannula (2 l/min). On the fourth day of hospitalization, COVID-19 treatment was stopped because her symptoms resolved, and the patient was discharged. 1 month later, follow up chest CT showed almost complete resolution of the lung infiltrates (Figure 2). Clinical course is illustrated in Figure 3.

## Discussion

To the best of our knowledge, this is the first reported case of a cancer patient who developed COVID-19 pneumonia while under treatment with nivolumab in combination with an investigational PEGylated IL-2. The exact beginning of COVID-19 pneumonia is vague due to overlap with nivolumab- and bempegaldesleukin-related fatigue, which the patient had experienced during the previous treatment cycles. Given that our patient presented 3 weeks after nivolumab/bempegaldesleukin administration and treatment-related fatigue during the previous cycles lasted for no more than 7 days, we hypothesize that COVID-19 was at least of 2-week duration before diagnosis. Although the patient reported only fatigue and had an oxygen saturation of 96% on room air at presentation, she was ultimately hospitalized and received oxygen supplementation and treatment with remdesivir and dexamethasone because she had dyspnea and oxygen desaturation upon minimal exertion as well as multiple lung infiltrates in chest CT. In addition, the patient had a high risk for deterioration (24%) and death (19%) according to the ISARIC 4C score [13]. Therefore the administration of the above agents might have significantly improved the outcome of our patient. Nevertheless, treatment with nivolumab and bempegaldesleukin might have contributed to the atypical initial presentation and protracted initial phase of the infection and did not lead to an adverse outcome.

Studies regarding the impact of ICIs in the course of the infection and its outcome in cancer patients have produced controversial results. Robilloti *et al.* showed that cancer patients treated with ICIs had a higher risk of hospitalization and severe respiratory illness [14]. However, several subsequent studies have shown that ICI use did not increase the COVID-19 severity among cancer patients [15–18]. Recently, Tan *et al.* used propensity score matching and no significant differences between the ICI-treated and non-ICI treated cohorts were recorded for mortality, hospitalization and emergency department visits [19]. Regarding melanoma, among 652 patients on active PD-1 inhibitor therapy in the registry of the German Working Group of Dermato-Oncology (ADOREG), only 13 patients were identified with COVID-19 and the disease was asymptomatic or mild in most cases [20]. It has been proposed that ICIs enhance the immune response against viral infections and may be protective against severe disease [21]. Interestingly, Diao *et al.* reported reduced numbers of CD4+ and CD8+ T-cells and increased levels of pro-inflammatory cytokines in COVID-19 patients, particularly in those requiring Intensive Care Unit admission. Furthermore, a progressive increase in the PD-1 and TIM3 expression was observed as patients deteriorated [22]. The above findings are suggestive of T-cell exhaustion in COVID-19 infection and suggest a potential role for ICIs in the treatment of patients with an ‘exhausted’ phenotype [23]. In a small, randomized, open-label, phase II trial in high-risk, hospitalized patients with COVID-19 pneumonia, the use of tocilizumab plus pembrolizumab versus standard of care reduced the hospitalization period [24]. Clinical trials of nivolumab in patients with COVID-19 have been planned (ClinicalTrials.gov Identifier: NCT04356508, NCT04343144 and NCT04413838). On the other hand, Rha *et al.* used MHC-I multimers and showed that PD-1+ SARS-CoV-2-specific CD8+ T cells are functionally active in terms of IFN-γ production, questioning whether cells are truly exhausted [25]. Moreover, there is concern that ICI use may induce cytokine-release syndrome [26]. Importantly, COVID-19 may occur concurrently with or may exacerbate immune-related (IR)-pneumonitis, leading to poor prognosis [14,26]. We cannot rule out concurrent COVID-19 and IR-pneumonitis in our patient on the basis of imaging findings alone. However, she improved with a short (4-day) course of corticosteroid treatment and did not relapse after dexamethasone discontinuation, which favors the COVID-19 pneumonia diagnosis as IR-pneumonitis requires prolonged corticosteroid treatment.

The influence of the administration of NKTR-214 on COVID-19 clinical course is currently unknown. Significantly higher levels of IL-2 were induced in patients with asymptomatic or mild disease compared to those with moderate and severe disease [27]. Shi *et al.* reported low levels of IL-2 in plasma and low expression of IL-2 receptor (IL-2R) in peripheral blood mononuclear cells (PBMCs) of critical COVID-19 patients, which may result in the remarkable decrease of CD8+ T-cells and lymphocytes in critical patients with COVID-19 pneumonia, and proposed that the progressive decrease of IL-2 in plasma may be a warning factor of disease deterioration [28]. Immune cell profiling analysis predicted that IL-2 may be beneficial for the recovery of COVID-19 patients [29]. Notably, IL-2 dysregulation is a characteristic of advanced age and might possibly contribute to the unfavorable outcome in the elderly. The age-related reduced production of IL-2 diminishes the ability of naive T-cells to differentiate into effector-cells, changes that impact on the intensity and duration of immune responses [30]. Furthermore, IL-2 induces cytotoxic activity and killing properties of natural killer (NK) cells, a process that is decreased in the elderly [30]. Zhu *et al.* reported a statistically significant increase in the lymphocyte count and a non-statistically significant decrease in CRP levels in patients treated with recombinant human IL-2 (rIL-2) compared with the control group [31]. Currently, the probable beneficial effect of bempegaldesleukin in COVID-19 is investigated in a phase Ib clinical trial (www.ClinicalTrial.gov Identifier: NCT04646044).

## Conclusions

Our case suggests that cancer patients treated with nivolumab and bempegaldesleukin do not seem to have adverse outcomes due to COVID-19 pneumonia. The strength of this report is that it describes a unique case of a COVID-19 pneumonia survivor treated with nivolumab and bempegaldesleukin for melanoma providing detailed clinical, laboratory and imaging data that may be useful to clinicians until experience in the management of such patients expands. The description of a single case, the lack of documentation of the exact beginning of COVID-19 pneumonia, the uncertainty about the exact cause of pneumonia, as well as the potential beneficial effect of dexamethasone and remdesivir are the main limitations of the current report. Nevertheless, this case gave us the opportunity to discuss the possible mechanisms of action of ICIs and IL-2 analogs in COVID-19 and we believe that it may alert clinicians to collect similar data and possibly clarify the issues raised. Further information is needed  to assess the effect of these agents in the outcome of cancer patients with COVID-19. Moreover, with the devastating impact of COVID-19 and the paucity of highly effective treatments, the results of currently ongoing clinical trials of ICIs and/or bempegaldesleukin in patients with COVID-19 are eagerly awaited.

**Summary points**

- We present the first case of COVID-19 pneumonia in an elderly melanoma patient treated with nivolumab and bempegaldesleukin.
- The administration of the combination of nivolumab plus bempegaldesleukin in an elderly melanoma patient did not result in an adverse outcome of COVID-19 pneumonia.
- Preclinical data suggest that the use of immune checkpoint inhibitors and bempegaldesleukin may modify the immune response to COVID-19 and may confer a beneficial effect against the infection.
- Ongoing clinical trials are evaluating immune checkpoint inhibitors and bempegaldesleukin in the treatment of COVID-19.
